# A Charging Algorithm for the Wireless Rechargeable Sensor Network with Imperfect Charging Channel and Finite Energy Storage

**DOI:** 10.3390/s19183887

**Published:** 2019-09-09

**Authors:** Mengqiu Tian, Wanguo Jiao, Jiaming Liu, Siyuan Ma

**Affiliations:** The College of Information Science and Technology, Nanjing Forestry University, Nanjing 210037, China; mqtian96@njfu.edu.cn (M.T.); cgyzmmmmyyy@njfu.edu.cn (S.M.)

**Keywords:** wireless charging, wireless sensor network, energy efficiency, mobile wireless charging car

## Abstract

Recently, wireless energy transfer technology becomes a popular way to address energy shortage in wireless sensor networks. The capacity of the mobile wireless charging car (WCV) and the wireless channel between the WCV and the sensor are two important factors influencing the energy efficiency of the wireless sensor network, which has not been well considered. In this paper, we study the energy efficiency of a wireless rechargeable sensor network charged by a finite capacity WCV through an imperfect wireless channel. To estimate the energy efficiency, we first propose a new metric named waste rate, which is defined as a function of the charging channel quality. Then, energy efficiency optimization is modeled as minimizing the waste rate. Through optimizing the distance between the WCV and sensor nodes, the set of optimal charging sensor nodes is obtained. By using the Hamiltonian circle, the nearest neighbor algorithm is proposed to find the traveling path of the WCV. Furthermore, to avoid the untimely death of sensor nodes and the coverage hole, an extended node dynamic replacement strategy is proposed. The simulation results show that the proposed method can reduce the waste rate and the total charging time; i.e., the sum of traveling time and charging delay can be significantly reduced, which indicates that the proposed algorithm can improve the energy efficiency of the network.

## 1. Introduction

### 1.1. Background and Motivation

Wireless sensor networks (WSNs) have a wide range of applications, such as military surveillance, environmental monitoring, disaster relief, smart home, etc. [1,2,3]. In the traditional wireless sensor network, the energy of the sensor node is provided by the battery. However, the capacity of the battery is limited by the size of the node, and the available energy of the sensor node is extremely insufficient, which greatly limits the application of the WSNs [4]. At the same time, the sensor node is often not easily accessible, and the cost of replacing the battery is often large, even is impossible sometimes. In order to make wireless sensor networks more practical, there are many works on the self-sustainability of the wireless sensor network [5,6]. The rechargeable sensor network offers a promising opportunity for the WSN [7,8,9].

In the rechargeable sensor network, many kinds of energy from the environment are used by the wireless sensor nodes, such as heat energy, solar energy, wind energy, and so on. In Reference [10], authors assume that sensors can harvest energy from natural energy source during the working period. Furthermore, Reference [11] considers the spatiotemporally coupled constraint in wireless rechargeable sensor networks (WRSNs) and proposes a distributed algorithm to get the optimal sampling rate to maximize network utility. By considering the time-varying recharging rate caused by the unreliable natural energy resource, an effective algorithm is proposed in Reference [12] to maintain the battery at the desired target level through optimizing the sampling rate and end-to-end routing path.

However, the natural energy often varies with the time and environment and the variation is unpredictable and uncontrollable, and sensor nodes are in the risk of unstable and insufficient energy supply. Different from the energy-harvesting technique, wireless energy transfer technology with magnetic resonance coupling [13] or electromagnetic effect [14] can provide a stable energy supply, which is a promising solution for extending the lifetime of wireless sensor networks. The result in Reference [15] shows that the wireless energy transfer technology is not only efficient but also immune to its surrounding environment. Industry research further demonstrates that it is possible to transfer 60 W of power over a distance of up to two to three feet with an energy transfer efficiency of 75% [16]. In the wireless rechargeable sensor network (WRSN), a wireless energy transfer technology realized by a mobile wireless charging vehicle (WCV) is applied to power sensor nodes. The results show that the lifetime of the WRSN is extremely extended [17]. The adoption of the WCV can provide high and stable charging rates to sensors. Thus, the cost of sensors and the complexity of energy management in WRSN is significantly reduced.

In this paper, we study the use of the WCV to replenish energy to sensor nodes in WRSNs. Some works have been done on the wireless sensor network with wireless energy transfer technology. In References [18,19,20], solutions for mobile charger scheduling and charging infrastructure deployment of rechargeable nodes are proposed. In Reference [21], the author assumes that the power of each charger is adjustable, and then finds a charger placement and a corresponding power allocation. Authors in Reference [22] study an on-demand energy replenishment problem and formulate it as an optimization problem with an objective of maximizing the number of charged sensors per tour. In Reference [23], the author assumes that each sensor node can be partially charged so that more sensor nodes can be charged by the WCV before their energy is depleted. Besides, the WCV not only serves as an energy transmitter to charge the static sensor nodes but also serves as a data collector in some works, such as [24,25,26,27]. Different from References [24,25,26] which focus on extending network lifetime, Reference [27] considers the problem of scheduling minimum mobile devices to periodically charge and collect data from sensors. The traveling path planning of the WCV is an important factor in the performance of WRSNs. Reference [28] aims to minimize the traveling cost of the WCV based on the energy monitoring and reporting protocols. In Reference [29], the author first analyzes the optimization opportunity and then proposes a novel charging strategy by modeling the problem as traveling salesman problem (TSP) with neighborhood. The model is able to exploit the wireless charging ability and reduce the moving delay of the charger at the same time. In Reference [30], the association between the WCV charging cycle and operational lifetime of sensor nodes is considered. A novel periodic charging algorithm is proposed which jointly considers charging tour planning and the WCV depot positioning. As the wireless energy transferring is over the wireless channel, the charging efficiency is sensitive to the Charging Channel Quality (CCQ). However, this factor has not been considered in the above works.

The distance between nodes is one of important factors influencing the CCQ. In Reference [31], the influence of the distance is considered. However, authors assumed that the capacity of the WCV is infinite, which is impossible in many cases in real life. In this paper, the WRSN is charged by a WCV which has limited capacity, at the same time, the influence of the CCQ on the energy replenishment of the sensor node is also considered. As the capacity of the WCV is finite, how to efficiently utilize the limited energy is important. To estimate the energy efficiency, we propose a metric named waste rate. Then, an algorithm is proposed to minimize the waste rate through finding optimal charging sensor nodes.

### 1.2. Contribution

As the WCV has limited capacity, minimizing waste rate is an important and realistic problem in the WRSN. To address this problem, through finding optimal sensor nodes charged by the WCV, the energy waste rate is reduced. The main contribution can be summarized as follows.

First, we take the limited capacity of WCV and the channel quality between the WCV and rechargeable sensor nodes into consideration and model the minimizing waste rate as an optimization problem. Then, we propose a method to find the optimal charging sensor nodes and use the nearest neighbor algorithm to get the traveling path of WCV. Furthermore, we also propose an extended node dynamic replacement strategy, which can avoid the death of uncharged sensor nodes and coverage holes. The experimental results demonstrate that our proposed solution can effectively reduce the waste rate and the total charging time comparing with the baseline scheme.

### 1.3. Paper Organization

The remaining sections are organized as follows. Section 2 introduces the system model in detail. In Section 3, we formulate the problem and provide a solving method. An extended node dynamic replacement strategy (ENDRS) is also proposed to avoid the death of uncharged nodes in Section 3. The simulation result is provided in Section 4. At last, we conclude the whole paper in Section 5. The main notations used in our paper are listed in Table 1.

## 2. System Model

### 2.1. Network Model

In this paper, we consider a set of sensor nodes, which is denoted by V, distributed over a limited two-dimensional area. The two-dimensional coordinate of sensor node $v_{i}\in V$ is $\left(x_{i},y_{i}\right)$. In the sensor network, there is a fixed base station *S*, which is also a sink node used to collect sensing data. We assume that the energy of *S* is unlimited comparing to sensor nodes. The set **E** constitutes the edges between two nodes which are in the transmission range of each other. Each sensor node $v_{i}$ is equipped with a battery whose capacity is $E_{\max}$. The initial energy level of sensor node $v_{i}$ is $E_{i,0}$ and $E_{i,0}=E_{\max}$. Notation $E_{i,t}$ denotes the residual energy of node $v_{i}$ at time t, and $E_{i,t}=E_{i,0}$ when t=0. The minimum energy level for regular operations is $E_{\min}$, hence the sensor nodes would stop working if the residual energy is lower than $E_{\min}$.

The energy consumed by sensor node $v_{i}$ can be divided into three parts: $e_{i,t}$, $e_{i,s}$, and $e_{i,r}$, which represent the energy consumed by sensor node $v_{i}$ in sending data, sensing data, and receiving data during [0,t], respectively. Among them, $e_{i,t}$ and $e_{i,r}$ include: <1> The energy consumed by receiving and sending data from its own nodes; <2> As the network works in the multi-hop pattern, some energy is used to receive and transmit data from neighboring sensor nodes. The energy consumption rate of the sensor node $v_{i}$ is defined as the energy consumed by the sensor node $v_{i}$ within unit time. The energy consumption rate of the sensor node $v_{i}$ before time *t* is denoted as $\varepsilon_{i,t}$, which can be calculated as

(1) $$\varepsilon_{i,t}=\frac{e_{i,t}+e_{i,r}+e_{i,s}}{t}$$

The operation of the WCV in the network is introduced in the following. The WCV with limited capacity leaves from the base station and charges the optimal sensor nodes which are selected by our proposed algorithm one by one. After charging these sensor nodes, it would back to the base station, and the traveling path of the WCV forms a cycle path. 

Notation $P_{i,c}$ represents the charging rate of the WCV to sensor node $v_{i}$, and the charging delay $T_{i,d}$ is the time that the WCV charges the sensor node $v_{i}$, which is defined as

(2) $$T_{i,d}=\frac{E_{i,0}-E_{i,t}-T_{i,d}*\varepsilon_{i,t}}{P_{i,c}}$$

Let $T_{(i-1),i}$ denote the total charging time from the moment that the WCV leaves sensor node $v_{i-1}$ to the moment that the WCV leaves sensor node $v_{i}$, which is expressed as

(3) $$T_{(i-1),i}=T_{i,d}+T_{travel(i-1\to i)}=T_{i,d}+\frac{h_{i}-h_{i-1}}{V_{c}},$$

where $T_{travel(i-1\to i)}$ is the time spent by the WCV from sensor node $v_{i-1}$ to the sensor node $v_{i}$, which is a function of the distance and the velocity. We use $h_{i}=(x_{i},y_{i})$ to denote the coordinate of sensor node $v_{i}$, and $h_{i}-h_{i-1}$ to denote the horizontal distance between sensor node $v_{i-1}$ and sensor node $v_{i}$, which is defined by Euclidean distance. Hence, $h_{i}-h_{i-1}=\sqrt{{\left(x_{i}-x_{i-1}\right)}^{2}+{\left(y_{i}-y_{i-1}\right)}^{2}}$. The WCV moves with a constant velocity which is denoted by $V_{c}$. According to Reference [32], we let $V_{c}$ = 5 m/s in the simulation.

In practical, due to the limited capacity of the WCV, the WCV may not be able to meet the charging requirement of all sensor nodes. Thus, choosing proper sensor nodes to be charged by the WCV is important. The CCQ is an important factor which influences the charging quality from the WCV to sensor node $v_{i}$. A good charging strategy should make the WCV with limited capacity charge more sensor nodes with better CCQ. To better measure the energy efficiency of the WCV, we innovatively propose the concept of waste rate, which also can be used in maximizing the efficiency of wireless charging at each node.

To support wireless power transmission, we assume that a receiving coil is installed on each sensor node. The CCQ between the sensor node $v_{i}$ and the WCV is related to many factors, such as the distance between the sensor node and the WCV, the noise, obstacle, and so on. In this paper, to simplify the model, we assume that the CCQ is only related to the distance. The waste rate is defined as the energy loss of the WCV to the sensor node when the CCQ is determined by the distance. The waste rate is an increasing function of distance. With the increase of distance, the waste rate increases exponentially [33]. Let *D* denote the charge range of the WCV.

### 2.2. Cellular Structure

We assume that the wireless rechargeable sensor area is a two-dimensional plane. For better zoning, we partition the two-dimensional plane into many hexagonal cells. Under the cellular structure, we denote $d_{i}$ as the distance from sensor node $v_{i}$ to its cell center. The sensor nodes in a cell constitute a cluster. In the remaining paper, we use the cluster to represent the cell and sensor nodes in the cell. The number of sensor nodes in a cluster is a random variable which is no less than 1. Some sensor nodes will send the charging requests to the base station which contains $N_{Rc}$ and $E_{c-i,t}$, where $N_{Rc}$ is the number of sensor nodes which send the charging request in the $c^{th}$ cluster, and $0\leq N_{Rc}\leq \rho$, ρ is the maximum number of sensor nodes in the $c^{th}$ cluster; $E_{c-i,t}$ is the residual energy of the sensor node $v_{i}$ which is in the $c^{th}$ cluster at time t. According to the residual energy of the sensor node which sends charging request in cluster, we divide these sensor nodes into three types: a, b, and *k*, where *a* represents the sensor nodes whose residual energy is greater than $E_{\min}$ and less than $0.1*E_{i,0}$ at time t, b represents the sensor nodes whose residual energy is greater than $0.1*E_{i,0}$ and less than $0.2*E_{i,0}$ at time t, and k represents the sensor nodes whose residual energy is greater than $0.2*E_{i,0}$ and less than $0.3*E_{i,0}$ at time t. The number of different types of sensor nodes in the $c^{th}$ cluster is denoted as $a_{c}$, $b_{c}$, and $k_{c}$ respectively. When the residual energy is larger than $0.3*E_{i,0}$, the sensor node does not send the charging request, thus $N_{Rc}=a_{c}+b_{c}+k_{c}$.

We assume that when the WCV reaches the charging working place, each sensor node can send its own CCQ to the WCV. After the WCV arrives in the cluster, it can stop and charge at any position. Therefore, there are infinite stopping points in the cluster, which leads to high complexity of finding the optimal stopping position. Since the size of the cell is small, the difference in the traveling cost of the WCV caused by different stopping points is very small. To simplify the model, we assume that the stopping point of the WCV is the center of the cell.

### 2.3. Limited Rechargeable Clusters

In our paper, we consider the limited capacity of the WCV, the WCV may not have enough energy to charge all sensor nodes which send charging requests in the clusters. Combining the cellular structure, we define the priority level of each cluster and use $weight_{c}$ to denote the weight value of the $c^{th}$ cluster. The priority of the $c^{th}$ cluster $weight_{c}$ is defined as

(4) $$weight_{c}=\omega N_{Rc}+\chi [\alpha a_{c}+\beta b_{c}+\gamma k_{c}],$$

where *ω*, *χ*, *α*, *β*, and *γ* are harmonic coefficients, which satisfy 

(5) ω+χ=1

(6) α+β+γ=1

From Equation (4), it can be found that different design goals can be achieved through adjusting the value of harmonic coefficients during cluster selection. When ω has a larger value, the number of sensor nodes which send charging requests is a main influence of cluster selection. When χ is larger, the distribution of residual energy becomes more uniform. The values of α, β, and γ mainly affect the preference of three types of sensor nodes. For example, when α is larger, we prefer to choose the cluster with more nodes of type a.

Clusters sending charging requests are sequentially stored in set A according to weight values. $A=\left\{c_{1},c_{2},\cdots ,c_{Num}\right\}$, where Num represents the number of clusters sending charging requests. Due to the limited capacity of the WCV, not all required charging clusters can be charged in a charging cycle. Therefore, the cluster with larger weight value should be selected first. This means that clusters with less energy and more sensor nodes sending charging requests often have a higher charging priority. At the same time, we assume that the WCV can only charge one sensor node in a cluster. This means that the number of selected charging clusters equals to the number of optimal charging sensor nodes. The selected cluster is stored in set $B=\left\{c_{1},c_{2},\cdots ,c_{N_{c}}\right\}$, where $N_{c}$ the number of selected charging clusters. Obviously, $N_{c}\leq Num$. The WCV will charge optimal sensor nodes in these clusters according to the Hamiltonian circle. The number of selected charging clusters is related to $T_{p,\max}$, which is the maximum time used by the WCV in a charging cycle. The relation between charging time $T_{(i-1),i}$ and the maximum time $T_{p,\max}$ is 

(7) $$\sum_{i=1}^{N_{c}}T_{(i-1),i}\leq T_{p,\max}.$$

When the traveling path is determined, the traveling time of the WCV is constant which is expressed by $N_{c}T_{travel}$ where $T_{travel}$ is the average value of $T_{travel(i-1\to i)}$. According to Equation (2), the charging time is proportional to the residual energy of the charged sensor node. Hence, when the remaining energy of all sensor nodes sending charging requests in the cluster is $0.3*E_{i,0}$, the total charging value gets the maximum. When the remaining energy of all sensor nodes sending charging requests in the cluster is the threshold $E_{\min}$, the total charging time is the minimum value of $\sum_{i=1}^{N_{c}}T_{(i-1),i}$. Thus, the range of the charging time can be expressed as 

(8) $$N_{c}(\frac{0.7*E_{i,0}+T_{i,d}*\varepsilon_{i,t}}{P_{i,c}}+T_{travel})\leq \sum_{i=1}^{N_{c}}T_{(i-1),i}\leq N_{c}(\frac{E_{i,0}+T_{i,d}*\varepsilon_{i,t}}{P_{i,c}}+T_{travel})$$

By transforming Equation (8), the range of $N_{C}$ can be obtained as

(9) $$\frac{\sum_{i=1}^{N_{c}}T_{(i-1),i}}{\left(\frac{E_{i,0}+T_{i,d}*\varepsilon_{i,t}}{P_{i,c}}+T_{travel}\right)}\leq N_{c}\leq \frac{\sum_{i=1}^{N_{c}}T_{(i-1),i}}{\left(\frac{0.7*E_{i,0}+T_{i,d}*\varepsilon_{i,t}}{P_{i,c}}+T_{travel}\right)}$$

According to the relationship between $T_{(i-1),i}$ and $T_{p,\max}$ in Equation (7), we derive the upper bound of the number of selected charging clusters, which is expressed as 

(10) $$N_{c}\leq \frac{T_{p,\max}}{\left(\frac{0.7*E_{i,0}+T_{i,d}*\varepsilon_{i,t}}{P_{i,c}}+T_{travel}\right)}$$

Furthermore, we can also use the capacity of the WCV, $T_{i,d}$, and the initial energy of the sensor node to calculate $N_{C}$ which is given as

(11) $$\frac{B_{c,\max}}{E_{i,0}+T_{i,d}*\varepsilon_{i,t}+E_{travel(i-1\to i)}}\leq N_{c}\leq \frac{B_{c,\max}}{0.7*E_{i,0}+T_{i,d}*\varepsilon_{i,t}+E_{travel(i-1\to i)}},$$

where $B_{c,\max}$ is the capacity of the WCV.

**Figure 1:**
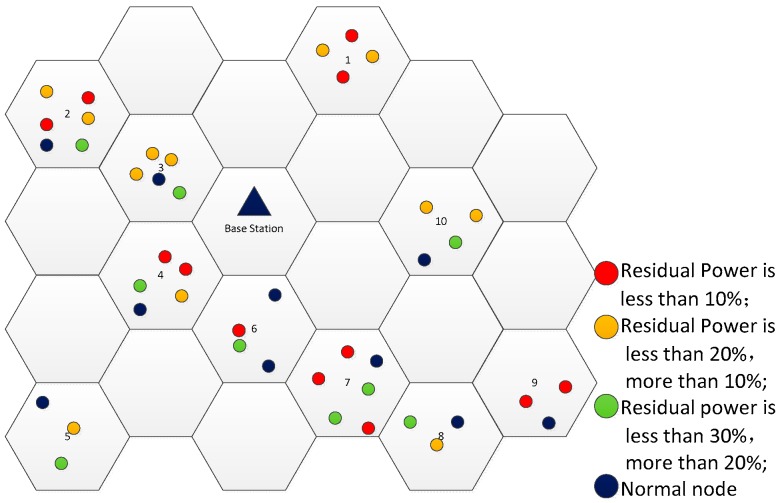
Sample diagram of prior charging clusters.

To demonstrate the above calculation, here is an example shown in Figure 1. It can be found that the number of clusters sending charging requests is 10, e.g., $N_{Rc}=10$.

As shown in Figure 1, each cluster has a random number of some sensor nodes and the number is in the range of [1,6]. According to the energy setting, in the $4^{th}$ cluster, $a_{4}=2$, $b_{4}=1$, $k_{4}=1$. Let ω=0.2, χ=0.8, α=0.7, β=0.2, and γ=0.1, respectively. Using Equation (4), we can obtain the weight value of $4^{th}$ cluster: $weight_{4}$ is 2.16. Similarly, the weight values of other clusters can be calculated, which are given in Table 2.

According to the weight value of each cluster, the clusters sending charging requests are sorted as follows: $c_{7}>c_{2}>c_{1}>c_{4}>c_{9}>c_{3}>c_{6}>c_{10}>c_{8}>c_{5}$. That is, $A{=\{c}_{7}{,c}_{2}{,c}_{1}{,c}_{4}{,c}_{9}{,c}_{3}{,c}_{6}{,c}_{10}{,c}_{8}{,c}_{5}\}$. Then, we use Equation (10) to find the prior charging clusters when $N_{C}$ is assumed to be 5, and obtain set $B=\left\{c_{7},c_{2},c_{1},c_{4},c_{9}\right\}$. Therefore, the WCV will charge the optimal sensor nodes in the 7th cluster, the 2th cluster, the 1th cluster, the 4th cluster, and the 9th cluster firstly.

**Figure 2:**
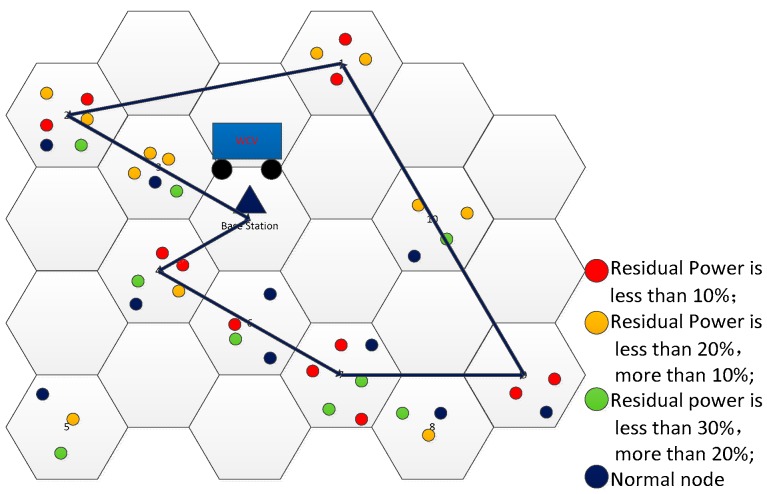
Diagram of the charging tour of the wireless charging vehicle (WCV).

As the capacity of the WCV is limited, the traveling path should be designed to reduce unnecessary energy waste on the journey. We make the WCV to charge along the Hamilton circle. The schematic diagram is shown in Figure 2.

The WCV starts from the base station and charges sensor nodes according to the Hamilton circle. In this paper, we use the nearest neighbor algorithm to get the optimal charging tour.

## 3. Problem Formulation and Solution

### 3.1. Problem Analysis

As discussed in Section 2, the CCQ between the WCV and the sensor node is only related to the distance. The waste rate $P_{i,waste}$ at sensor node $v_{i}$ is defined as

(12) $$P_{i,waste}=\frac{U_{i,full}-U_{i,in}}{U_{i,full}},$$

where $U_{i,full}$ is the output charging power from the WCV and $U_{i,in}$ is the charging power received by sensor node $v_{i}$. The charging power $U_{i,in}$ can be expressed as

(13) $$U_{i,in}=\varpi (d_{i})*U_{i,full},$$

where $\varpi (d_{i})$ is a function of distance $d_{i}$ and $0<\varpi (d_{i})<1$, which is used to expressed the CCQ. 

By combining Equations (12) and (13), the relation between the waste rate and $\varpi (d_{i})$ is 

(14) $$P_{i,waste}=1-\varpi (d_{i}).$$

According to Equation (14), it can be found that the energy waste rate is closely related to the CCQ. Because the CCQ between the WCV and the sensor node is only related to the distance, the waste rate $P_{i,waste}$ depends on the distance. This means that energy efficiency is related to the distance. As improving energy efficiency can be realized through minimizing the energy waste rate, we optimize the distance between the WCV and the sensor node to minimize the energy waste rate. For the optimal object, we propose an algorithm to find the optimal traveling path of the WCV and optimal charging sensor nodes.

Due to the large number of sensor nodes, to estimate the waste rate of all selected sensor nodes, we define the average of energy waste rate of optimal sensor nodes as

(15) $$P_{waste}=\frac{1}{N_{c}}\sum_{i=1}^{N_{c}}P_{i,waste}<1.$$

According to Equations (14) and (15), the waste rate $P_{i,waste}$ at sensor node $v_{i}$ and the average waste rate of sensor nodes $P_{waste}$ are less than 1.

Based on the above analysis, we model minimizing energy waste rate as an optimal problem, which is given by:

(16a) $$\min\;P_{waste}=\frac{1}{N_{c}}\sum_{i=1}^{N_{c}}P_{i,waste}$$

(16b) $$s.t.\;\frac{1}{N_{c}}\sum_{i=1}^{N_{c}}P_{i,waste}<1$$

(16c) $$\sum_{i=1}^{N_{c}}T_{(i-1),i}\leq T_{p,\max}$$

(16d) $$P_{i,waste}=1-\varpi (d_{i})<1$$

The constraint in Equation (16b) indicates that the average of waste rate should be less than 1 while Equation (16c) indicates that the total charging time is less than the maximum time of the WCV in a charging cycle.

As the different traveling path affect the energy consumed by the WCV moving from one sensor node to another one, the number of chargeable sensor nodes is influenced by the traveling path of the WCV. Besides, according to constraint (16d), the waste rate depends on the distance between the sensor node and the WCV. Hence, the minimum value can be achieved by choosing the optimal sensor node. To solve the problem in Equation (16a), we proposed two algorithms to plan the traveling path of the WCV and select the optimally charged sensor node, respectively. 

### 3.2. The Traveling Path Planning

According to the above discussion, the different traveling paths results in different traveling distances, the time and the energy spent on different traveling paths will be different, thus the optimal traveling path of the WCV should be proposed to reduce the unnecessary energy waste. Though the path planning has been researched in some previous works, such as Reference [17], the energy waste suffered by the traveling path of the WCV has not been considered. The traveling path not only affects the energy waste rate but also has an influence on the charging time for each node. In order to avoid unnecessary energy waste, we optimize the traveling path of the WCV to minimize the energy waste rate. We assume that the WCV travels along the Hamiltonian circle, which is a traveling salesman problem (TSP) [34]. Analogous details of proof process could be found in Reference [17].

To solve the required Hamiltonian circle, the nearest neighbor algorithm is proposed. The detail of the nearest neighbor algorithm is provided in Algorithm 1.

**Algorithm 1.** The nearest neighbor algorithm.
**Input:**
$[x_{i},y_{i}],0\leq i\leq N_{c}$
**Output:** The traveling path of the WCV
1:Calculate the distance between each priority cluster and the base station, and sort the distance in ascending order. Select the cluster $c_{count}$ which is closest to the base station. The initial value of *count* is 1.2:Calculate the distance of the remaining clusters from the cluster $c_{count}$, then select the cluster $c_{count+1}$ which is closest to the cluster $c_{count}$, count=count+1.3:Repeat step 2 until $count=N_{c}$, $N_{c}$ is the number of selected clusters.4:The WCV drive from the cluster $c_{N_{c}}$ to base station.


**Figure 3:**
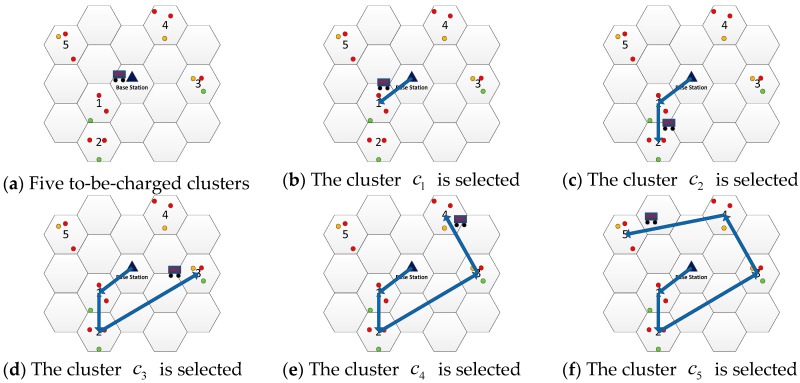
An example of Algorithm 1.

To illustrate Algorithm 1, we use an example shown in Figure 3. Assume that there are five to-be-charged clusters in the network at some time point (see Figure 3a). First, we calculate the distance between each cluster and the base station, and sort the distance in ascending order which is $d_{1}{<d}_{2}{<d}_{3}{<d}_{4}{<d}_{5}$. The nearest cluster $c_{1}$ is first selected (see Figure 3b). Then we calculate the distance of the remaining clusters from the cluster $c_{1}$, and the order is $d_{2}{<d}_{3}{<d}_{4}{<d}_{5}$. The cluster $c_{2}$ is selected since it is closest to the cluster $c_{1}$ (see Figure 3c). Similarly, the remaining clusters are selected one by one (see Figure 3d,e). Finally, after charging cluster $c_{5}$ (see Figure 3f), the WCV will return to the base station to recharge itself. A charging tour according to the nearest neighbor algorithm is formed.

### 3.3. The Selection of Optimal Charging Nodes

According to the above discussion, the energy efficiency is related to the distance. As improving energy efficiency can be realized through minimizing the energy waste rate, the distance between the WCV and the sensor node is optimized to reduce the energy waste rate. To minimize the energy waste, based on the results of Algorithm 1, we propose a node selection algorithm shown in Algorithm 2.

**Algorithm 2.** Procedure of solution for our problem.**Input:** Two-dimensional coordinates of sensor nodes $\left[x_{i},y_{i}\right]$, the distance between each sensor node $v_{i}$ in the $c_{th}$ priority cluster and the WCV $d_{i}$, the number of selected charging nodes $N_{c}$, the density of nodes ρ, initial energy of each sensor node $E_{i,0}$ and the remaining energy $E_{i,t}$, the speed of the WCV $V_{c}$.**Output:** Energy average waste rate $P_{waste}$, the total charging time $T_{per}$, the traveling path of the WCV.
1:Partition the two-dimensional plane with hexagonal cells. When the residual energy of nodes $E_{i,t}$ reaches the charging threshold, each node sends a charging request in units of clusters. The clusters are sorted according to the weight values.$weight_{c}$ which is obtained by Equation (4), $N_{c}$ prior charging clusters are selected and then $N_{c}$ optimal sensor nodes are selected for charging.2:Sort the waste rate $P_{i,waste}$ between the WCV and sensor node $v_{i}$ after the WCV enters the cluster which is determined by the distance between the WCV and the sensor nodes $d_{i}$.3:Find the optimal charging sensor nodes by considering their waste rate, which means that the sensor nodes with better CCQ are selected for charging.4:Calculate the corresponding charging time $T_{per}$ and achieve the traveling path of the WCV according to Algorithm 1.


**Figure 4:**
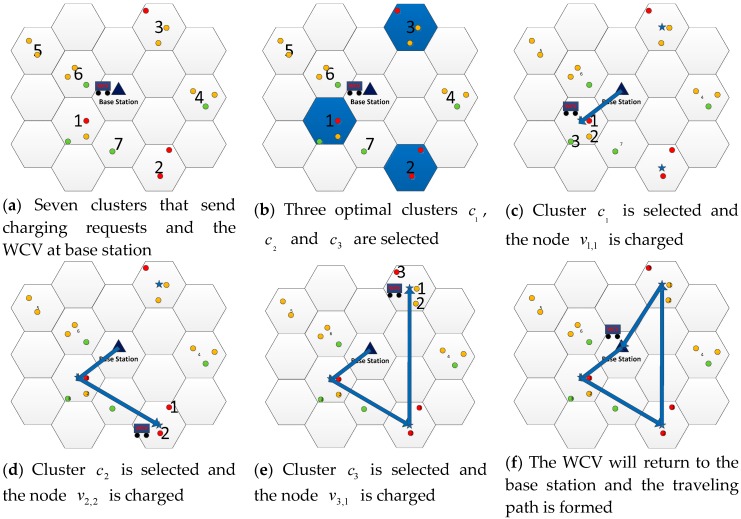
An example of the execution of Algorithm 2.

To illustrate the execution of Algorithm 2, we use a similar example shown in Figure 4. Assume that there are some clusters sending charging requests in the network at some time point (see Figure 4a). The clusters are sorted according to the weight value of the $c^{th}$ cluster $weight_{c}$ by Equation (4), which is $c_{7}<c_{5}<c_{{}_{4}}<c_{6}<c_{1}<c_{3}<c_{2}$. We assume $N_{c}=3$ which can be obtained by Algorithm 1 and Equation (10), it means the WCV can only charge 3 clusters (see Figure 4b). According to Algorithm 1, the WCV will charge cluster $c_{1}$ first, after entering cluster $c_{1}$, there are three to-be-charged sensor nodes $v_{1,1}$, $v_{1,2}$ and $v_{1,3}$. Sort the waste rate $P_{i,waste}$ which is determined by the distance between the WCV and sensor nodes $d_{i}$, and the order is $P_{1,waste}<P_{2,waste}<P_{3,waste}$. As sensor node *v*_1,1_ has better CCQ, it is selected to be charged (see Figure 4c). Then, the WCV leaves cluster $c_{1}$ to cluster $c_{2}$, the order of waste rate $P_{i,waste}$ of sensor nodes $v_{2,1}$ and $v_{2,2}$ is $P_{2,waste}<P_{1,waste}$. Thus, the node $v_{2,2}$ is selected (see Figure 4d). Similarly, the optimal charging sensor node $v_{3,1}$ in cluster $c_{3}$ can be found (see Figure 4e). Finally, the WCV will return to the base station and the charging round of the WCV is finished (see Figure 4f).

First, we prove Theorem 1 to illustrate sensor nodes obtained by Algorithm 2 are optimal sensor nodes. Then, we study the complexity of Algorithm 2.

**Theorem** **1.** *Through Algorithm 2, we can get the optimal charging sensor nodes which can minimize the energy waste rate.*

**Proof.**  Assumption: Given an optimal solution $\psi^{*}=(V^{*},T^{*},P_{waste}^{*})$, where some of the sensor nodes selected are not optimal. Then we could construct a new solution $\overset{\wedge }{\psi }=(\overset{\wedge }{V},\overset{\wedge }{T},\overset{\wedge }{P_{waste}})$ where all the selected sensor nodes are optimal. With the assumption, $V^{*}$ in $\psi^{*}$ is the set of these selected charging sensor nodes, while some of nodes such as $V_{i}^{*}$ and $V_{j}^{*}$ are not optimal, and we assume that the $N_{c}$ in $\psi^{*}$ and $\overset{\wedge }{\psi }$ is same, that is, $N_{c}^{*}=\overset{\wedge }{N_{c}}$. Then the new solution $\overset{\wedge }{\psi }$ could be constructed as follows. Let $\overset{\wedge }{V}$ is the set of selected optimal charging sensor nodes and $\overset{\wedge }{T}$ is the related total charging time per cycle. Because all nodes in $\overset{\wedge }{V}$ is optimal, we can get $\overset{\wedge }{P_{i,waste}}\leq P_{i,waste}^{*}$, $\overset{\wedge }{P_{j,waste}}\leq P_{j,waste}^{*}$, from Equation (16d), where the $\overset{\wedge }{P_{i,waste}}$, $\overset{\wedge }{P_{j,waste}}$ and $P_{i,waste}^{*}$, $P_{j,waste}^{*}$ represent the waste rate of sensor nodes $v_{i}$, $v_{j}$ in the $\psi^{*}$ and $\overset{\wedge }{\psi }$, respectively. For $N_{c}^{*}=\overset{\wedge }{N_{c}}$, we can get that in the $\psi^{*}$ and $\overset{\wedge }{\psi }$, the average waste rate of sensor nodes is different, that is, $\overset{\wedge }{P_{waste}}\leq P_{waste}^{*}$. At the same time, the difference in waste rate results in the difference of charging efficiency, thus results in the difference of total charging time with different solutions. From Equation (2), we can get $\overset{\wedge }{T}\leq T^{*}$. Then it can be found that the solution $\overset{\wedge }{\psi }$ could provide an improved objective. □

Next, we show that the solution $\overset{\wedge }{\psi }$ is feasible for our optimal problem. To verify feasibility, we need to show that $\overset{\wedge }{\psi }$ satisfy constraints Equations (16b–d). Since the $\overset{\wedge }{\psi }$ is a feasible solution for our problem, it should satisfy the constraints Equations (16b–d). For $\overset{\wedge }{T}\leq T^{*}$, from Equation (16c), $T^{*}<T_{p,\max}$, we can get $\overset{\wedge }{T}\leq T_{p,\max}$, which could satisfy Equation (16c). For ${\overset{\wedge }{P}}_{\mathrm{waste}}\leq P_{\mathrm{waste}}^{*}$, it can be straightforwardly found that ${\overset{\wedge }{P}}_{\mathrm{waste}}\leq P_{\mathrm{waste}}^{*}<1$, which satisfy Equation (16b). We assume that sensor nodes $v_{i}$ in the $\psi^{*}$ is not optimal, thus ${\overset{\wedge }{P_{i,}}}_{\mathrm{waste}}\leq P_{i,\mathrm{waste}}^{*}$. Because $P_{i,\mathrm{waste}}^{*}<1$, we can get ${\overset{\wedge }{P}}_{i,\mathrm{waste}}\leq P_{i,\mathrm{waste}}^{*}<1$, which satisfies Equation (16d).

Thus, the solution $\overset{\wedge }{\psi }=(\overset{\wedge }{V},\overset{\wedge }{T},{\overset{\wedge }{P}}_{waste})$ is a feasible solution, which could provide an improved objective comparing to the optimal solution $\psi^{*}$.

Theorem 2 shows an analysis of the time complexity of our algorithm.

**Theorem** **2.** *The time complexity of the Algorithm 2 is bounded in $O({\left(\frac{l}{R_{c}}\right)}^{2}+n+N_{c}^{2})$, where *l*, *n*, *N_c_* denote the side length of the sensor field, the number of deployed sensors, and the number of optimal charging sensor nodes, respectively*.

**Proof.**  Because *l* is the length of the sensor field, the division of the sensor field requires at most $O({\left(\frac{l}{R_{c}}\right)}^{2})$. *n* sensor nodes are considered for calculating related CCQ for the selection of the optimal charging sensor nodes, the complex of the nodes is *O*(*n*). *N_c_* selected optimal charging sensor nodes are considered in turn to obtain the traveling path of the WCV. For example, at first, there are *N_c_* optimal charging sensor nodes are considered to calculate the traveling path of the WCV, the complex is *O*(*N_c_*).Then, there are *N_c_* − 1 selected optimal charging sensor nodes are considered to calculate the traveling path of the WCV, the complex is *O*(*N_c_* − 1)., at the end of the algorithm, there is only one sensor node, the complex is *O*(1),. Thus, the complex of the optimal charging sensor nodes is $O(N_{c}+N_{c}*(N_{c}-1)/2)$, that is, $O{\left(N_{c}\right)}^{2}$. So, the complexity of the Algorithm 2 is bounded in $O({\left(\frac{l}{R_{c}}\right)}^{2}+n+N_{c}^{2})$. This completes the proof. □

### 3.4. Extend Node Dynamic Replacement Strategy

According to the description of Algorithm 2, the optimal charging sensor node in a cluster may not be the one with the least energy. The sensor node whose residual energy is close to the threshold $E_{\min}$ is named the life-critical sensor node. Since the WCV does not charge the life-critical sensor node timely, the residual energy will be exhausted in a short time and the sensor node will be dead due to the shortage of energy. To avoid the death of sensor nodes whose charging requests are not satisfied, we also proposed the extended node dynamic replacement strategy (ENDRS).

According to the above discussion, the optimal charging sensor node in a cluster may not be the one with the least energy. To avoid the death of nodes in this charging cycle, the life-critical sensor node will go to sleep and its work will be transferred to the optimal charging sensor node. In the next round of the charging cycle, the sleep node will be considered according to the ENDRS which is based on Algorithm 1 and Algorithm 2. The detail of the ENDRS can be described as follows.

**Figure 5:**
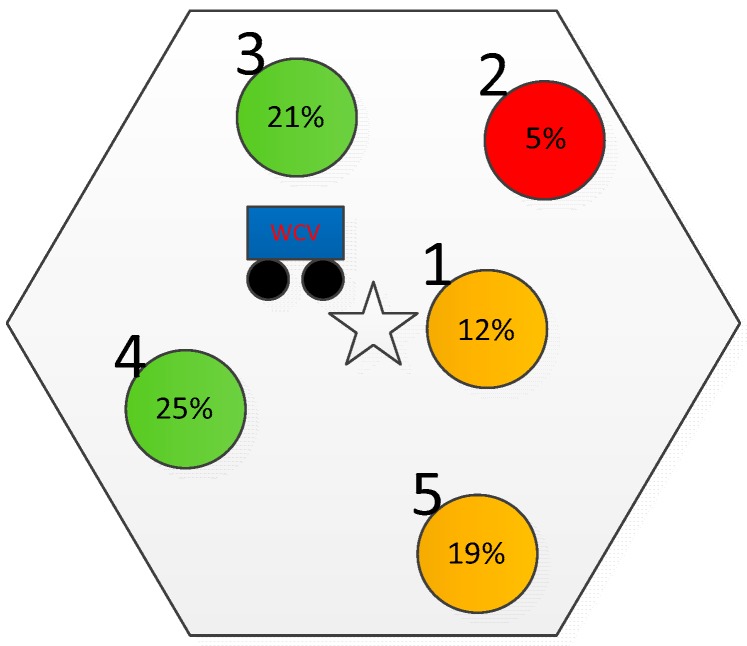
An example to introduce the node replacement strategy.

After choosing optimal charging nodes, clusters which have sleep sensor nodes are recorded. In the next charging round, the values of harmonic coefficients in Equation (4) during prior cluster selection are changed. This change should ensure that the cluster with sleep nodes has more chance to be selected. After the WCV enters a cluster with the sleep node, the tradeoff between the charging efficiency and the lifetime of the sensor node is considered during the optimal charging sensor node selection. We define the weight of optimal charging sensor node selection as $\zeta d_{i}+(1-\zeta )E_{c-i,t}$, where different goals can be achieved through adjusting the value of the harmonic coefficient ζ. When (1−ζ) has a larger value, the residual energy is a main influence of selection. This means that the WCV prefers to choose the life-critical node to ensure the connectivity of network. To further illustrate the ENDRS strategy, we provide an example in Figure 5.

As shown in Figure 5, there is a cluster constituting of 5 sensor nodes. According to Algorithm 2, the WCV selects node 1 which has 12% energy, instead of node 2 which has 5% energy. The node 2 will die soon, which may lead to coverage holes in this area. Moreover, the connectivity and stability of the network may be degraded. If the ENDRS strategy is adopted, node 2 will go to sleep and the work of node 2 is transferred to node 1. In the next round of the charging cycle, node 2 will have chance to be charged by adjusting the harmonic coefficient and the weight in the ENDRS, which ensures the connectivity and stability of the network.

## 4. Simulation Evaluation

In this section, some simulation results are provided to evaluate the performance of the proposed algorithm. Through studying these results, the impact of the network parameter on the network performance is obtained.

### 4.1. Parameter Setting

**Figure 6:**
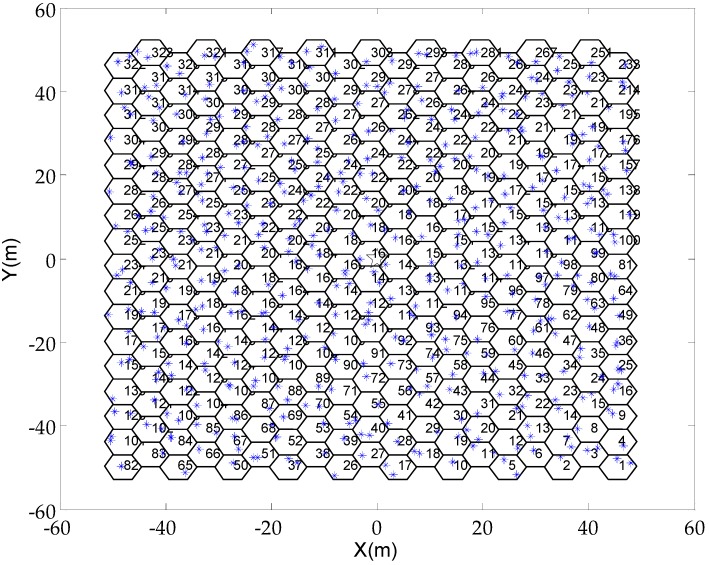
The scenario diagram.

We assume that each cell contains a random number of sensor nodes, and define ρ as the density of the sensor node. The density of the sensor node is defined as the maximum number of sensor nodes in a cell, e.g., ρ≥1. When the density of the sensor node increases, the total number of sensor nodes in the network also increases. By using MATLAB software, a network scenario in which the density of the sensor node is 2 are generated, which is shown in Figure 6.

**Figure 7:**
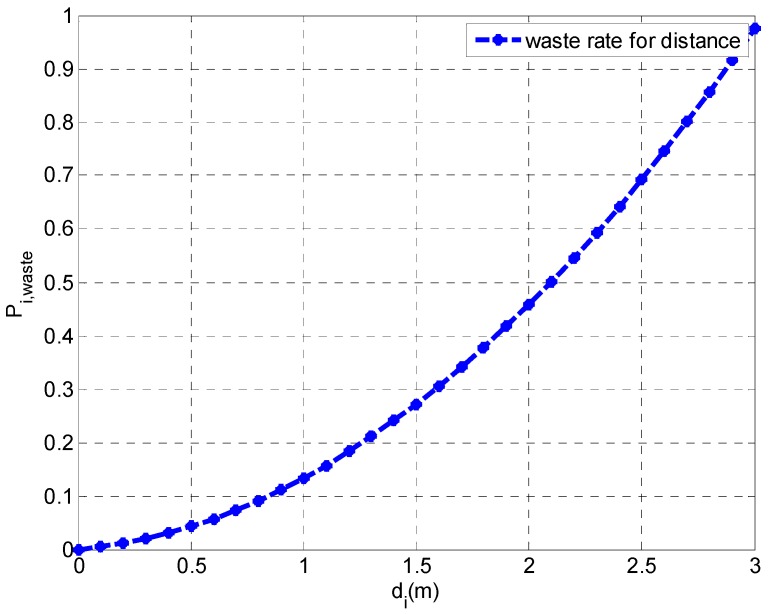
Energy waste rate varying with the distance.

In order to prevent the WCV from running out of energy and being unable to drive back to the base station, we set that the WCV can charge for 25 nodes in a charging cycle. The size of each hexagon cell is 3 m. The consumption rate of each sensor node is set as $rand(1)/10^{11}$ J, and the initial energy of each sensor node is $E_{0}=0.02$ J. The WCV moves at a constant speed of $V_{c}$ = 5 m/s with an energy consumption rate of $E_{v}=1/10^{12}$ J/m. The base station is the origin (0,0). According to the curve fitting experiment in Reference [35], we define $P_{i,waste}=-0.095812*d_{i}{}^{2}-0.03771*d_{i}+1.0$. When the distance of charging is more than 3 m, we assume that the charging efficiency of the WCV is 0, that is, the waste rate $P_{i,waste}$ is 1. According to Equation (14), we draw the relation between the energy waste rate and the distance, which is given in Figure 7.

The result in Figure 7 indicates that the energy waste rate increases with the increasing of the distance, which is consistent with Equation (14). In order to decrease the waste rate, the distance between the sensor node and the WCV should be short as much as possible. Furthermore, this one-to-one correspondence relationship makes the distance be replaced by the waste rare during parameter setting of the simulation. 

### 4.2. Results and Analysis

**Figure 8:**
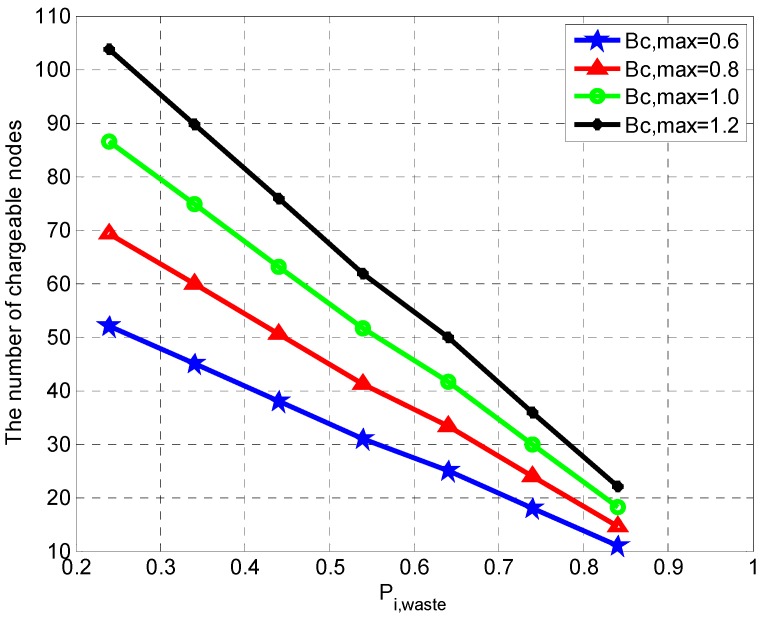
The number of chargeable nodes under different energy waste rate.

Under the same constant density of the sensor node, we study the impacts of energy waste rate and the capacity of WCV on the number of chargeable sensor nodes in a charging cycle. We first observe the number of chargeable sensor nodes when the energy waste rates are 0.24, 0.34, 0.44, 0.54, 0.64, 0.74, and 0.84, respectively. The results are shown in Figure 8 when the capacity of WCV $B_{c,\max}$ is 0.6 J, 0.8 J, 1.0 J, and 1.2 J, respectively.

From Figure 8, it can be found that the number of chargeable sensor nodes increases with the decreasing of the energy waste rate or the increasing of the capacity of the WCV. This phenomenon can be explained by using Equation (11). Take the right side of the equation, the number of chargeable sensor nodes is $N_{c}=\frac{B_{c}^{*}{}_{,\max}}{0.7*E_{i,0}+T_{i,d}*\rho_{i,t}+E_{travel(i-1\to i)}}$, where $B_{{}_{c,\max}}^{*}=(1-P_{i,waste})*B_{c,\max}$ represents the energy actually used. The original formula is changed into: $N_{c}=\psi *(1-P_{i,waste})$, where $\psi =\frac{B_{c,\max}}{0.7*E_{i,0}+T_{i,d}*\varepsilon_{i,t}+E_{travel(i-1\to i)}}$.

**Figure 9:**
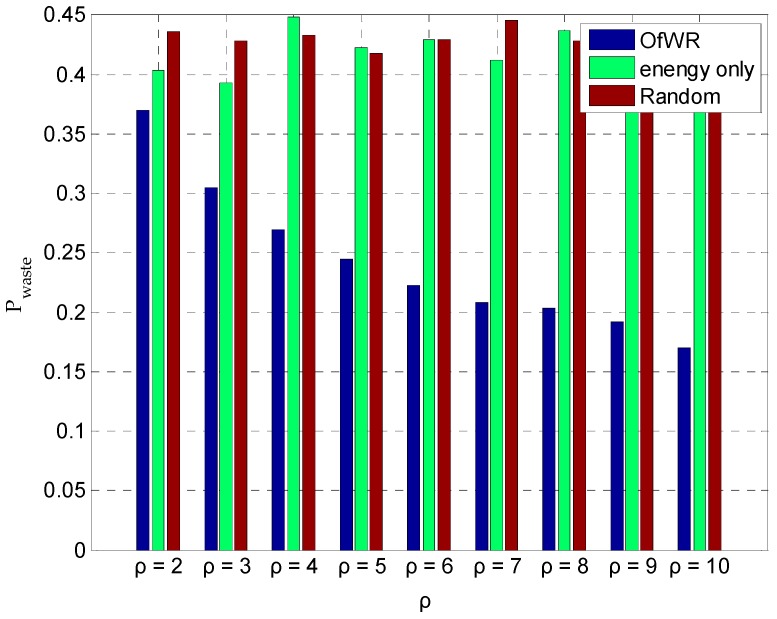
The comparison of the energy waste rate.

The density of the sensor node ρ has a great impact on the number of sensor nodes in the work. Next, we study the impact of the density of the sensor node on the average energy waste rate $P_{waste}$. In the simulation, the density of the sensor node ρ is 2, 3, 4, 5, 6, 7, 8, 9, and 10, respectively. The comparison results of three different algorithms is given in Figure 9. In Figure 9, the proposed algorithm is expressed by “OfWR”, “energy only” represents the algorithm only considers the impact of residual energy of sensor nodes, and “Random” denotes sensor nodes are randomly choose to be charged according to the Random algorithm. In the energy only algorithm, the WCV will choose the node which has the least residual energy to charge. In the Random algorithm, the WCV randomly choose one of sensor nodes which send the charging request. From the results in Figure 9, it can be found that the energy waste of the OfWR is the least and the decreases with the increasing of the density of the sensor node. Hence, the proposed algorithm can achieve better performance in the energy waste rate, especially when the density of the sensor nodes is higher. It indicates that our proposed algorithm can achieve higher energy efficiency in the network with a large density of the sensor node.

**Figure 10:**
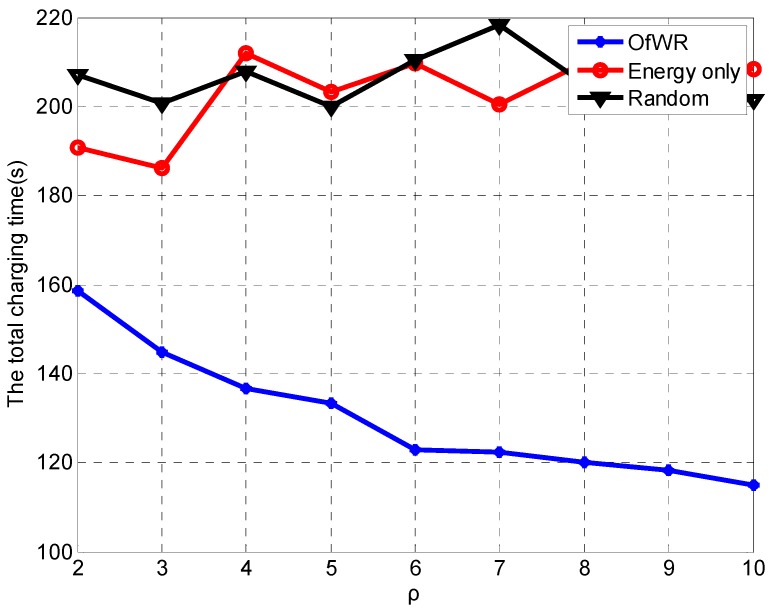
The comparison of the total charging time.

Furthermore, we compare the charging time when the WCV can charge for 25 sensor nodes in a round. The variation of total charging time in terms of the density of the sensor nodes is shown in Figure 10.

The results in Figure 10 shows that the total charging time of the proposed algorithm is less than half time of other algorithms when the density of the sensor node is 2. With the increasing of ρ, the difference between the proposed algorithm and the other algorithms is enlarged. It indicates that our proposed algorithm can reduce the charging time in the network with a large density of the sensor node.

**Figure 11:**
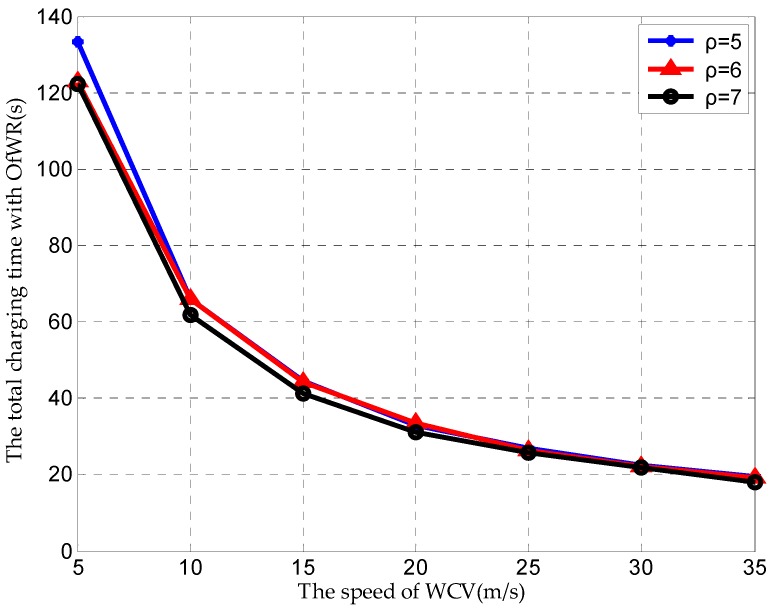
The total charging time varying with the speed of WCV.

Finally, we observe the total charging time varying with the speed of the WCV under different densities of the sensor node, and the result is in Figure 11. The result in Figure 11 shows that the total charging time decreases with the increasing of the speed of the WCV, and the difference among different densities of the sensor node is not obvious. This result indicates that the total charging time can be decreased through increasing the speed of the WCV, and when the speed of the WCV is more than 20 m/s, the proposed algorithm can achieve the almost same performance of the charging time under a smaller density of sensor node.

**Figure 12:**
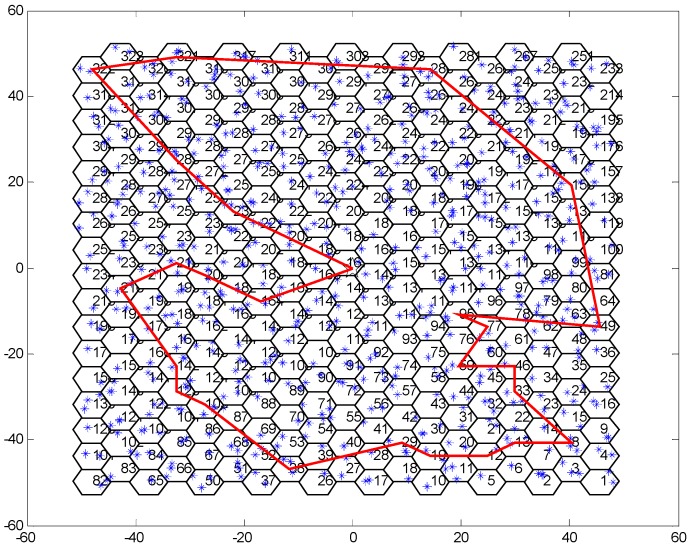
The traveling path of the WCV.

According to the analysis in Section 3, to optimize the traveling path of the WCV, the WCV should travel along the Hamiltonian circle, which is a traveling salesman problem. By using the nearest neighbor algorithm, the traveling path of the WCV can be found. In the simulation, the traveling path of the WCV obtained by Algorithm 1 is shown in Figure 12. The result in Figure 12 demonstrates that the WCV moves according to the Hamilton circle.

## 5. Conclusions

In this paper, we consider that the mobile WCV charges sensor nodes in clusters to achieve a self-sustainable rechargeable wireless sensor network. Due to the limited capacity of WCV, it may be impossible to charge all sensor nodes which send the charging request within a charging round. To maximize energy efficiency, we model an optimization problem with the object of minimizing the average energy waste rate when the limited capacity of the WCV and the imperfect CCQ are considered. The problem is solved by using two proposed algorithms: Algorithm 1 and Algorithm 2. Algorithm 1 is used to attain the traveling path of WCV while Algorithm 2 is used to find the optimally charged sensor node. An extended node dynamic replacement strategy is further proposed to avoid the death of uncharged life-critical sensor nodes. The simulation result shows that the proposed algorithms can reduce the energy waste rate and the total charging time, especially when the density of the sensor node is large.

## Figures and Tables

**Table 1:** Symbol definitions.

Symbols	Definition
$E_{i,0}$	The initial energy level of sensor node $v_{i}$
$E_{i,t}$	The residual energy of node $v_{i}$ at time
$E_{\max}$	The capacity of the sensor node $v_{i}$
$E_{\min}$	The minimum energy required by the regular operation
$\varepsilon_{i,t}$	The energy consumption rate of sensor node $v_{i}$ before time t
$T_{i,d}$	The charging time of sensor node $v_{i}$
$T_{(i-1),i}$	The total charging time from sensor node $v_{i-1}$ to sensor node $v_{i}$
$T_{travel(i-1\to i)}$	The time spent by the WCV from sensor node $v_{i-1}$ to sensor node $v_{i}$
$T_{per}$	The total charging time during a charging circle
$V_{c}$	The speed of the WCV
*N_c_*	The number of selected charging clusters
$N_{Rc}$	The number of sensor nodes which send the charging request in the $c^{th}$ cluster
Num	The number of clusters which send the charging request
$T_{p,\max}$	The maximum time of the WCV in a charging round
$E_{c-i,t}$	The residual energy of sensor node $v_{i}$ in the $c^{th}$ cluster at time t
$B_{c,\max}$	The capacity of the WCV
$P_{i,waste}$	The waste rate of sensor node $v_{i}$
ρ	The density of the sensor node

**Table 2:** The weight value of each cluster.

Cluster Number	1	2	3	4	5	6	7	8	9	10
weight	2.24	2.52	1.36	2.16	0.64	1.04	2.84	0.64	1.52	1
